# Crystal structure of HutZ, a heme storage protein from *Vibrio cholerae*: A structural mismatch observed in the region of high sequence conservation

**DOI:** 10.1186/1472-6807-12-23

**Published:** 2012-09-26

**Authors:** Xiuhua Liu, Jing Gong, Tiandi Wei, Zhi Wang, Qian Du, Deyu Zhu, Yan Huang, Sujuan Xu, Lichuan Gu

**Affiliations:** 1 State Key Laboratory of Microbial Technology, School of Life Sciences, Shandong University, Jinan, 250100, China; 2 Cancer Research Center, School of Medicine, Shandong University, Jinan, 250012, China; 3 College of Life Sciences, Hebei University, Baoding, 071002, China

**Keywords:** HutZ, Heme-binding, Crystal structure, Homology modeling

## Abstract

**Background:**

HutZ is the sole heme storage protein identified in the pathogenic bacterium *Vibrio cholerae* and is required for optimal heme utilization. However, no heme oxygenase activity has been observed with this protein. Thus far, HutZ’s structure and heme-binding mechanism are unknown.

**Results:**

We report the first crystal structure of HutZ in a homodimer determined at 2.0 Å resolution. The HutZ structure adopted a typical split-barrel fold. Through a docking study and site-directed mutagenesis, a heme-binding model for the HutZ dimer is proposed. Very interestingly, structural superimposition of HutZ and its homologous protein HugZ, a heme oxygenase from *Helicobacter pylori*, exhibited a structural mismatch of one amino acid residue in β6 of HutZ, although residues involved in this region are highly conserved in both proteins. Derived homologous models of different single point variants with model evaluations suggested that Pro^140^ of HutZ, corresponding to Phe^215^ of HugZ, might have been the main contributor to the structural mismatch. This mismatch initiates more divergent structural characteristics towards their C-terminal regions, which are essential features for the heme-binding of HugZ as a heme oxygenase.

**Conclusions:**

HutZ’s deficiency in heme oxygenase activity might derive from its residue shift relative to the heme oxygenase HugZ. This residue shift also emphasized a limitation of the traditional template selection criterion for homology modeling.

## Background

Iron is an essential element for the Gram-negative pathogenic bacterium *Vibrio cholerae*. It plays important roles in the microbe’s survival and its ability to cause the diarrheal disease cholera of *V. cholerae*. Nevertheless, the concentration of free iron is extremely low in the environment as well as in the human hosts. Under iron starvation conditions, *V. cholerae* has evolved several high-affinity iron uptake systems [1]. Synthesis and secretion of the catechol-type siderophore vibriobactin is the main mechanism for obtaining iron [2]. Siderophores, such as schizokinen [3], enterobactin [4,5] and ferrichrome [6], produced by other microorganisms, can also be utilized by *V. cholerae*.

Heme, an excellent iron source in the environment and human hosts, can be used by *V. cholerae* in the free form or with heme-binding proteins [7,8]. It is first transported into the cell with the assistance of the corresponding TonB-dependent outer membrane receptors and ATP-binding cassette transporter system proteins [3,9], and then the iron released from heme by cytoplasmic heme oxygenase.

However, to date, no heme oxygenase has been reported in *V. cholerae*. BLAST searches against the NCBI database [10] have also returned no heme oxygenase homologues for *V. cholerae*. Therefore, the fate of heme after it enters *V. cholerae*’s cytoplasm remains mysterious, and little is known regarding which proteins contribute to heme utilization in the cytoplasm. In 2004, Wyckoff and coworkers identified in *V. cholerae* the heme-binding protein HutZ, a protein essential for the optimal utilization of heme as an iron source [11]. However, no heme oxygenase activity has been observed for this protein, which indicates that HutZ serves only as a heme storage protein [11]. NCBI BLAST searches have shown that HutZ belongs to the pyridoxine-5'-phosphate (PNP) oxidase-like superfamily and shares 35% sequence identity with the heme oxygenases HugZ from *Helicobacter pylori*[12,13] and ChuZ from *Campylobacter jejuni*[14]. Meanwhile, sequence comparisons have shown that HutZ shares low sequence identities (~10%) with other known representative heme oxygenases, such as HmuO from *Corynebacterium diphtheria*[15], HemO from *Neisseria meningitidis*[16], IsdG from Bacillus anthracis [17], and ChuS from *Escherichia coli* O157:H7 [18].

To gain further insight into the mechanism of the mysterious function of HutZ, this protein from *V. cholerae* (strain N16961) was overexpressed and its crystal structure determined at 2.0 Å resolution. The HutZ structure appeared to a typical split-barrel fold that is usually conserved in FMN-binding proteins. As heme did not cocrystallize with HutZ, molecular docking and site-directed mutation experiments were performed to investigate the interaction mechanism of HutZ and heme.

In the absence of experimentally solved protein structures, homology modeling is widely used to predict a structure based on one or more known structures of homologous proteins. Such a model is developed based on the general rule that similar protein sequences correspond to similar protein structures. Notably, in the comparison of the structures of HutZ and HugZ, a structural mismatch was identified in one amino acid residue after the corner of β6 of HutZ, where both protein sequences are identical except for two pairs of different amino acid residues. This observation suggested a potential hazard in the accuracy or application of the homology modeling method, in particular in the accuracy of the input sequence alignment. In the light of this, a series of homologous models were constructed to further verify the sequence-structure relationship between HutZ and HugZ and to explore functional implications from the HutZ structure. As ChuZ and HugZ share high sequence identity (53%) and structural similarity (root mean square deviation (RMSD) of 2.1 Å for the protein backbone), HugZ was employed as the representative heme oxygenase for homology modeling of different HutZ variants and for structural comparisons.

## Results

### The overall structure of HutZ

**Figure 1:**
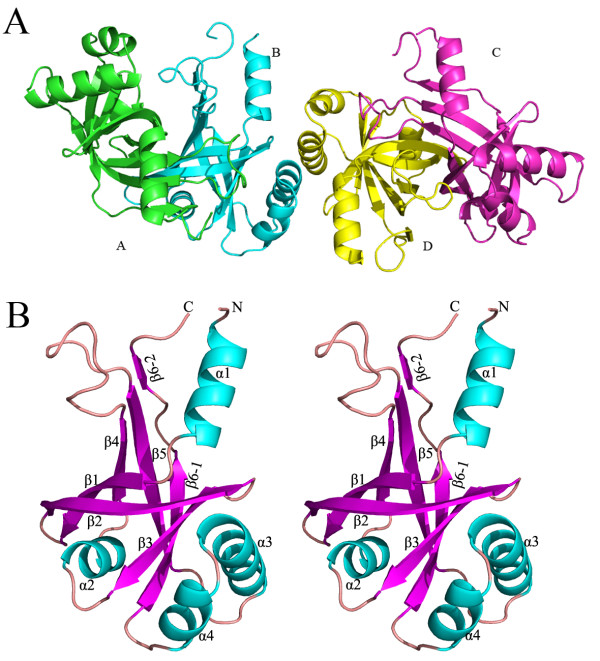
** Overall structure of HutZ(A) and stereoview ofHutZ monomer (B).** (**A**) There are two dimers in an asymmetric unit, termed A (green), B (cyan), C (magenta) and D (yellow) for clarification. (**B**) Rainbow-colored scheme representation of HutZ monomer. Four α-helices and six β-strands are labeled.

The structure of HutZ contains four monomer molecules (A, B, C and D) in an asymmetric unit that form two homodimers (AB and CD) (Figure 1A). Each monomer includes amino acid residues 13–150 of the entire HutZ protein (residues 1–176). Protein interface analysis with CCP4i showed that the interface area between monomers A and B was 1575.1 Å^2^ and 1569.4 Å^2^ for C and D, covering approximately 18% of the total solvent accessible surface area of 17208 Å^2^. This suggested that the homodimer forms, which represent the aggregation state of most split-barrel proteins in solution, were stable for HutZ. The existence of HutZ homodimers in solution was further confirmed by gel filtration chromatography on a Superdex-200 column (Additional file 1).

**Figure 2:**
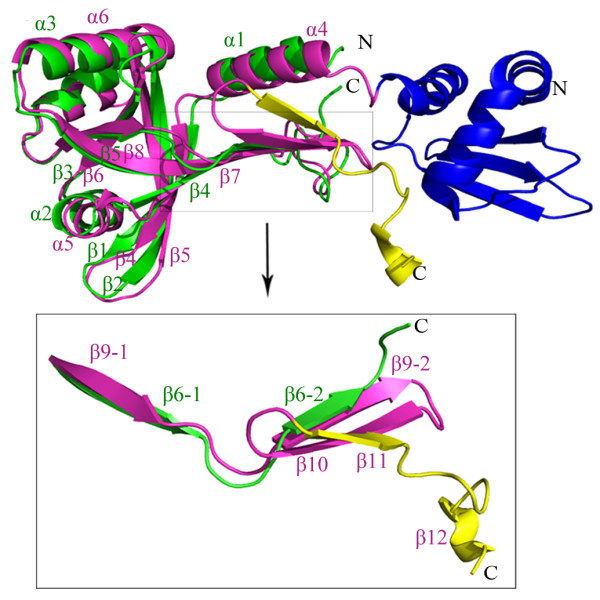
** Structure comparison of HutZand HugZ monomers.** Overall HutZ monomer folding (green) is very similar to the homologous protein HugZ (magenta). HugZ N-terminal domain which is absent in HutZ is labeled in blue, and HugZ variable C-terminal region is in yellow. Secondary structural assignment for HutZ was labeled in green and HugZ in magenta.

Although the HutZ homodimer adopted a typical split-barrel fold that is commonly conserved in FMN-binding proteins, no such activity was observed for HutZ (data not shown). Superimposition of both monomers from each dimer produces an RMSD of 0.294 Å for all corresponding Cα atoms. A HutZ monomer consisted of four α-helices interwoven with six β-strands (Figure 1B), a structure similar to the C-terminal domain of HugZ (PDB code: 3GAS) (Figure 2) [13]. Six antiparallel strands, β1-β6, produced a β-barrel of a Greek key topology (Additional file 2). Helices α1 and α2 were packed against the β-barrel and blocked each opening of the β-barrel. Helices α3 and α4 were loaded on one side of the β-barrel, with the HutZ dimer bound mainly through interactions of exposed β-barrel from each monomer. The two β-barrels were packed with each other side by side, bringing together surfaces distant from helices α3 and α4 on the loading side. 

### Heme-binding site

**Figure 3:**
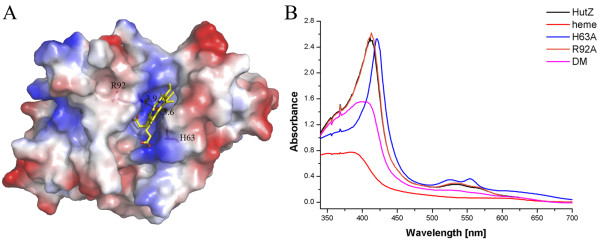
** Heme binding to HutZ.** (**A**) Docking result of HutZ dimer-heme complex. HutZ dimer is shown in surface charge representation, with positive charge in blue, and negative charge in red. Heme molecule is labeled in yellow stick, with iron atom in red sphere. His^63^ and Arg^92^ that coordinate with heme iron atom are represented in magenta stick. The distance (Å) between His^63^/Arg^92^ and iron atom is in black dash. (**B**) Absorbance spectra of HutZ and its mutants reconstituted with heme. DM: double mutant.

A number of approaches were attempted to produce crystals of HutZ-heme complex, but no crystals were obtained. In this case, we carried out molecular docking to investigate the interactions between heme and HutZ. There are two large clefts in the HutZ dimer interfaces that are postulated, due to similarities with HugZ, to be the heme-binding pocket. The first-ranked resulting model of HutZ dimer-heme complex showed that the main contributors to the heme coordination included His^63^ loaded at the helix α2 of one monomer and Arg^92^ originating from strand β5 of the other (Figure 3A). These two residues cooperatively coordinated the iron atom in the center of the heme molecule, with the distance between His^63^ and the iron atom at approximately 3.6 Å and between Arg^92^ and the iron atom at 3.9 Å. UV absorption spectral analysis on native and mutant forms of HutZ-heme complexes were performed to explore whether His^63^ and Arg^92^ were both necessary for heme-binding. The Soret peak for the native HutZ-heme complex was at 410 nm; for the single mutant H63A reconstituted with heme, it was slightly shifted to 420 nm, indicating that His^63^ slightly affected heme binding. For R92A, the Soret band was not altered in comparison with the native HutZ-heme complex (Figure 3B), suggesting that Arg^92^ did not affect heme binding. However, there was no characteristic Soret peak detected for the double point mutant H63A-R92A reconstituted with heme. The Soret peak data collected were from full length HutZ and truncated HutZ (data not shown for full length HutZ). These observations indicated that at least one of these residues, His^63^ and Arg^92^, must exist to effectively coordinate the iron atom in heme, and either of them is sufficient. Additionally, eleven residues from monomer A (Ser^42^, Tyr^43^, Pro^45^, Ser^58^, Ile^60^, Ala^61^, Arg^62^, Arg^65^, Leu^127^, Asp^132^ and Phe^133^) and three residues from monomer B (Phe^88^, Thr^94^ and Phe^145^) around the heme-binding cleft participated in stabilizing the heme. These residues were fully conserved or replaced with similar residues in comparison with the residues stabilizing the heme group in the HugZ-heme complex (Additional file 3).

### Structural comparison between HutZ and HugZ

**Figure 4:**
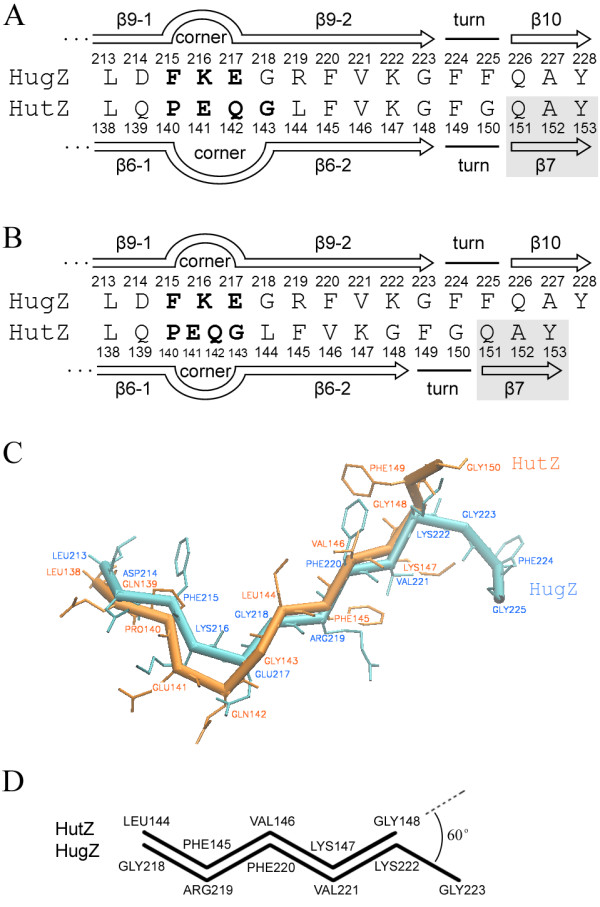
** Sequence and structural comparisonsof HutZ and HugZstructural shift regions.** (**A**) Sequence correspondence based on sequence alignment. Secondary structures are labeled above or below sequences. The shaded region represents the hypothetical β7 of HutZ, which does not exist in present crystal structure. (**B**) Sequence correspondence based on structure superimposition. Different from A, the four-residue-long corner of HutZ is aligned to three-residue-long corner of HugZ. Secondary structures are labeled above or below sequences. (**C**) Structural superimposition of HutZ and HugZ. Protein backbones are shown in trace representation with side chains in bond. (**D**) C-terminals of main chains of β6-2 and β9-2 point in different directions forming a 60° angle.

Structural superimposition revealed that the HutZ monomer shared high structural similarity with HugZ monomer (structure RMSD = 1.645 Å and sequence identity = 35%), which is a heme oxygenase from *Helicobacter pylori*[12,13]. Sequence alignment showed that the HugZ N-terminal domain (residues 1–80) was absent in HutZ, and the C-terminal loop (residues 238–249) of HugZ was variable in HutZ. In HutZ, the β6 contained a four-residue-long corner structure (Pro^140^-Gly^143^) that divides β6 into two segments (Figure 1B), designated as β6-1 (Phe^133^-Gln^139^) and β6-2 (Leu^144^-Gly^148^) respectively, while the corresponding β9 in HugZ is also divided into two segments β9-1 (Phe^208^-Asp^214^) and β9-2 (Gly^218^-Gly^223^) by a three-residue-long corner (Phe^215^-Glu^217^) (Figure 4A). The *Fo*_*Fc* omit map clearly revealed the positions of the four amino acid residues composing the corner of β6 in HutZ (Additional file 4). Very interestingly, although the segment Gly^143^-Tyr^153^ in HutZ was identical to the segment Gly^218^-Tyr^228^ in HugZ except for two pairs of amino acid residues (Arg^219^ of HutZ and Leu^144^ of HugZ, Phe^225^ of HutZ and Gly^150^ of HugZ) (Figure 4A), these residues did not match each other in one-to-one correspondence in the superimposition of three-dimensional structures (Figure 4B and C). Rather, β6-2 was shifted frontward by one amino acid residue relative to β9-2. For example, Phe^145^ of HutZ did not match Phe^220^ of HugZ, but corresponded to the preceding Arg^219^. This structural mismatch was substantially represented as changes in hydrogen bonding patterns between adjacent β-strands. Two adjacent β-strands form a hydrogen bond network in which the N-H groups or C = O groups of one strand establish hydrogen bonds with the C = O groups or N-H groups of another. Normally, there are two hydrogen bonds (N-H-O and O-H-N) on every second amino acid residue in one strand. In the HutZ and HugZ structures, the hydrogen bonding patterns were both regular on β6-1 and β9-1 (Additional file 5. A and B). However, in the four-residue-long corner of HutZ, no hydrogen bonds were detected, whereas in the three-residue-long corner of HugZ, two consecutive hydrogen bonds existed on Lys^216^ and Glu^217^. In this vein, after both corner regions, the correspondence between the hydrogen bond providing residues on β6-2 and β9-2 were not in line with that of their sequence alignment (Additional file 5. C). There was a mismatch of one amino acid residue. 

**Figure 5:**
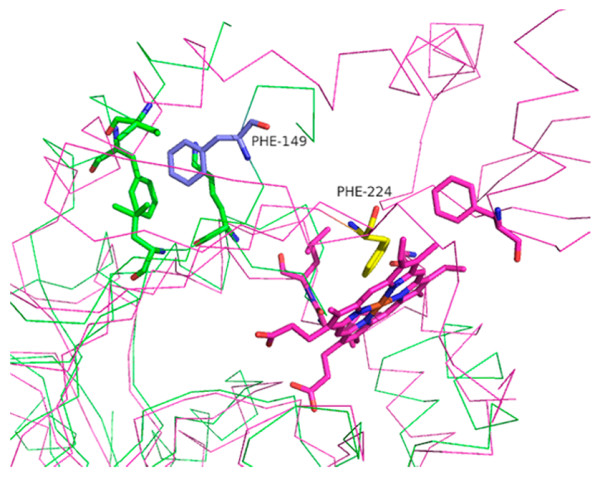
** Conformational comparison between Phe**^**149**^**of HutZ and thecorresponding residue Phe**^**224**^**of HugZ.** HutZ is displayed in green ribbon and HugZ in magenta ribbon. Phe^149^ is labeled as blue stick, and its side chain points to hydrophobic pocket formed by residues in green. Phe^224^ is in yellow stick, and its side chain points in opposite direction.

Due to this structural mismatch, the side chains of all residues involved in this region pointed to opposite directions in HutZ and HugZ (Figure 4C). The side chain of Phe^149^ in HutZ protruded into a hydrophobic pocket formed by Ile^18^, Phe^21^, Lys^91^ and Leu^93^, whereas the side chain of corresponding residue Phe^224^ in HugZ turns to another hydrophobic pocket on the opposite side (Figure 5). Starting from this pair of residues, the conformations of the succeeding residues become significantly different between HutZ and HugZ.

### Structural modeling of HutZ and its variants

Anfinsen’s dogma [19] states that a protein’s native structure is determined by its amino acid sequence and is a stable and kinetically accessible minimum of free energy. Accordingly, the local structural divergence between HutZ and HugZ should result from certain amino acid diversities. Therefore five homologous models for HutZ and its variants were constructed, and their rationalities were assessed to determine their essential residues. The compatibility of every amino acid with its conformation was assessed by ProQres, but the focus was on the β6 of HutZ and the corresponding regions in other models, where the structural mismatch occured.

**Figure 6:**
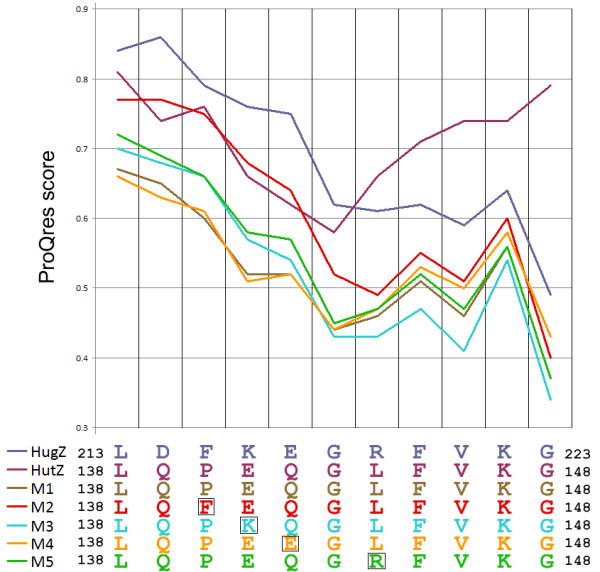
** Structure evaluations of crystalstructures and models.** ProQres score ranged from 0 for a random prediction to 1 for a perfect prediction. Boxed residues are single mutations in models.

The first model (M1) was generated for the native sequence of HutZ using the HugZ’s structure as the template. Thus, M1 possessed HutZ’s sequence and did not show a structural mismatch with HugZ. Structural evaluations showed that both crystal structures received higher scores than M1 (Figure 6). This result indicated that the present crystal structure, which possessed a structural mismatch to HugZ, better fitted the HutZ protein sequence than M1, which matched well the structure of HugZ; namely, the structural mismatch of the present crystal structure was reasonable. Simultaneously, the structure of HugZ also well adapted to the sequence of HugZ.

Next, the goal was to identify which residue was the main contributor to HutZ’s structural shift. From the beginning of the corner region (Pro^140^), where the structural mismatch begins, to Tyr^153^ of HutZ, there were a total of four different residue pairs between HutZ and HugZ (Figure 4A). These residues were very likely responsible for the structural shift and, thus, four single point variants (P140F, E141K, Q142E and L144R) for HutZ were designed and four models (M2-M5) generated for them, respectively (Figure 6). The mutated residues in HutZ were simply replaced with the corresponding residues of HugZ, and, as with M1, all variant models used the HugZ structure as a template, such that all of them were aligned with HugZ in one-to-one correspondence both on the sequence and structural levels. Structural evaluations of these models suggested three things (Figure 6). First, the two crystal structures were most favored by their sequences, as they generally received higher scores than the homologous models. Second, the score curves of the HugZ crystal structure and all homologous models exhibited similar shapes, which were obviously different from the HutZ crystal structure, because all models were constructed using the HugZ crystal structure as a template. Last, among all homologous models, M2 was evaluated as the best, while M1, M3, M4 and M5 were close to one another, suggesting that the sequence of the variant P140F better fitted the HugZ structure than those of other variants. In other words, although the residues Pro^140^, Glu^141^, Gln^142^ and Leu^144^ may together have contributed to the formation of HutZ’s structural shift, Pro^140^ played a more important role than other residues. Moreover, Pro^140^ was located at the very beginning of the corner region, where the structural shift also began, underlining the role of Pro^140^ in the local folding of HutZ β6.

## Discussion

HutZ, a unique heme storage protein identified in *V. cholerae*, is necessary for optimal heme utilization [11,20], but no heme oxygenase activity has been detected for HutZ. The crystal structure of the heme oxygenase HugZ shows that there are two symmetric active sites located at the HugZ dimmer interface, formed by the C-terminal region (β8-β11 and the C-terminal loop) from one monomer and α7 from the other [13]. The C-terminal loop functions as a flexible portion of the active site, which is supposed to keep the substrate heme molecule in proper conformation for the heme oxygenase activity as well as to close off the active pocket [13]. In the HutZ structure, the homologous heme-binding pocket involved β5 and β6, which corresponded with β8 and β9 in HugZ. The structure from β7 to the C-terminal loop, which corresponded to β10, β11 and the C-terminal loop in HugZ, was truncated in the present structure. Thus, it was unclear whether the missing portion participated in the heme-binding pocket or it resulted in the lack of HutZ enzymic activity. His^170^ from the C-terminal of HutZ, corresponds to the fully conserved His^245^ in HugZ that is responsible for the heme iron atom coordination [13]. Notably here, HutZ’s His^170^ did not contribute to iron coordination and enzymatic activity in HutZ as, when mutant H170A was reconstituted with heme, the Soret peak did not change in comparison with the native HutZ-heme complex (data not shown). This study of the structural mismatch between HutZ and HugZ provided an important clue for resolving this issue. Because the corner of β6 of HutZ includes one more amino acid residue than that of HugZ, HutZ’s β6-2 (five residues) ends one amino acid earlier than HugZ’s β9-2 (six residues). It is known that the torsion angle of N-Cα-C-N in the backbone of a β-strand is about 120°. Therefore, the main chains of the end residues of the two β-strands (β6-2 and β9-2) point in different directions to form a 60° angle (Figure 4D). This direction deviation led to an absolute mismatch between the structures of HutZ (Phe^149^ and Gly^150^) and HugZ (Gly^223^ and Phe^224^) \(Figure 4C), and might have led to even larger structural differences in their C-terminal regions (missing in the present structure). In this regard, it was speculated that the C-terminal loop, which is essential for HugZ enzymatic activity, might have turned away from the heme-binding pocket of HutZ and yielded the pocket more exposed than that of HugZ. This might then have resulted in low heme-binding affinity and a deficiency in HutZ enzymatic activity. In addition, the possibility was excluded here that truncating the protein resulted in a structural shift of β6-2 in HutZ for three reasons. First, HutZ was truncated here because of the twinning and fragile crystals of the full length protein. If the complete HutZ structure matched HugZ at β6-2 and the C-terminal loop, good full length crystals would have been obtained, as have been attained by Hu and coworkers [13]. Second, an extensive hydrogen bond network connected two parallel β-stands and maintained their stable conformations. Truncating the residues that follow β6-2 might not have provided enough energy to destroy the normal hydrogen bonding and rearrange them. And third, the hypothetical HutZ structure (M1) that matches HugZ at β6-2 evaluated even more poorly than the present truncated HutZ structure.

## Methods

### Protein expression, purification and site-directed mutagenesis

The *hutZ* gene was amplified from genomic DNA of *V. cholerae* and subcloned into the pET21b expression vector (Novagen, EMD Biosciences, Inc., Darmstadt, DE) between the NdeI and XhoI restriction cut sites. The N-terminal and C-terminal regions (residues 1–12 and 151–176, respectively) were removed during cloning, resulting in a construct of 13-150-His tagged fusion protein for further expression.

BL21 (DE3) cells containing plasmids for the recombinant HutZ protein were grown in L Broth media supplemented with 100 μg/mL ampicillin. Once the culture attained an OD_600_ of 1.0, the incubation temperature was decreased to 15°C, and protein expression induced by adding isopropyl β-D-1-thiogalactopyranoside to a final concentration of 0.10 mM. After 8 h of expression, the cells were harvested by centrifugation and the pellet resuspended in lysis buffer (15 mM Tris–HCl, pH 8.0, 100 mM NaCl, and 1 mM phenyl methane sulfonyl fluoride), and lysed by sonication. Cell debris was then removed by centrifugation at 28500 × g for 45 min. The resulting soluble fractions containing recombinant protein HutZ was loaded onto a Ni-chelating Sepharose affinity column (GE Healthcare, Buckinghamshire, UK) equilibrated with lysis buffer. The affinity column was washed extensively with lysis buffer and all proteins eluted with elution buffer (20 mM Tris–HCl, pH 8.0, 50 mM NaCl and 200 mM imidazole). The elution protein was further purified using an ion-exchange column (Source 15Q, GE Healthcare), conditioned with equilibration buffer (25 mM Tris–HCl, pH 8.0, 3 mM DTT), and eluted using a linear 150 mL gradient of 0–0.5 M NaCl. Finally, HutZ protein was purified using size exclusion chromatography (Superdex-200, GE Healthcare). Fractions were pooled according to protein purity monitored by SDS-PAGE, and the final protein concentration at 12 mg/mL. All mutant proteins were purified using the same procedure.

### Crystallization and data collection

HutZ was crystallized using the sitting drop vapor diffusion method at 20°C by mixing equal volumes of protein with reservoir solution containing 1.6 M Na/K phosphate and 0.1 M HEPES at pH 7.5. Because the crystals of full length HutZ were twinning and fragile and could not be used to obtain high resolution diffraction data, the protein was truncated to amino acid residues 13–150 to grow high quality crystals, which appeared about seven to ten d and reached full size in two wk. Diffraction data were collected at the Shanghai Sychrotron Radiation Facility beamline BL17U1. To prevent radiation damage, crystals were transferred to a cryoprotectant buffer containing 15% glycerol (v/v) plus reservoir buffer and then flash-cooled using a nitrogen stream, with the temperature around the crystals maintained at 100 K throughout data collection. Data sets were processed using the HKL2000 software suite [21]. The crystals belonged to the space group P4_1_ with four macromolecules in an asymmetric unit, with unit cell dimensions of a = b = 80.1 Å and c = 125.8 Å.

### Structure determination

HutZ’s structure was determined by molecular replacement with PHASER from CCP4i software, using the search model Alr5027 (PDB code: 1VL7) from *Nostoc sp.* with sequence identity of 43%. The atomic model was built using COOT [22] and refined using PHENIX [23]. Data collection and structure refinement statistics are shown in Table 1. All molecular graphics figures were generated with PyMol (http://www.pymol.org). 

**Table 1:** Statistics of crystallographic analysis

**Data collection**	
Space group	P4_1_
unit cell (Å)	a=b=80.1 c=125.8
Resolution (Å)	50–2.15
Completeness	95.5 (95.9)^a^
Redundancy	10.0 (7.8)
I/σ(I)	24.5 (7.2)
Rsym (%)^b^	10.4 (47.4)
Refinement	
Resolution	50-2.0
Rwork/Rfree (%)^c^	19.73/23. 51
RMSD	
Bond lengths (Å)	0.007
Bond angles (°)	1.070
Ramachandran plot (%)^d^	
Most favored (%)	92.9
Additionally allowed (%)	6.9
Generously allowed (%)	0.2
Disallowed (%)	0.0

^a^ Values in parentheses are for reflections in the highest resolution shell.

^b^*R*_sym_=∑_*hkl*_∑_*i*_|*I*(*hkl*)_*i*_ -<*I*(*hkl*)>|/∑_*hkl*_∑*I*<*I*(*hkl*)_*i*_>over *i* observations.

^c^ Value of *R*_free_ for 3.91% of randomly selected reflections excluded from refinement.

^d^ As defined in PROCHECK.

### Docking and homology modeling

AutoDock 4.2 [24] was used to perform flexible docking in the HutZ-heme complex. The heme was restricted within a grid box (50 × 50 × 30 points in dimension and 0.375 Å spacing) that enveloped the HutZ binding cleft, which was determined through similarities with the known crystal structure of the HugZ-heme complex. Docking searches were executed using the Lamarckian genetic algorithm and a maximum number of 25,000,000 energy evaluations. After docking searches were finished, the first-ranked model, based on binding energy, was selected as the final result from 50 candidate solutions.

A series of homologous models of HutZ and its variants were generated using the MODELLER 9.9 program package [25] with HugZ (PDB code: 3GAS) as a template. The input files for each target sequence were a pairwise sequence alignment (template and target) and the coordinate file of the template. The number of output models for each target was set to five, and other MODELLER options at default. The qualities of all resulting models were evaluated with ProQres [26], a neural-network based local protein model quality predictor, which analyzes the compatibility of every amino acid residue with its conformation in a three-dimensional model.

## Conclusions

We determined the crystal structure of HutZ from *V. cholerae* at 2.0 Å resolution and compared the structure with that of its homologous protein HugZ. The structural mismatch between HutZ and HugZ presented a rare case in which the structural alignment was not in accordance with the sequence alignment for two highly similar proteins (structure RMSD = 1.645 Ã and sequence identity = 35%). This observation suggested a potential hazard in the assumed accuracy of template selection of the traditional homology modeling method. If a homologous model of HutZ is constructed using the default sequence alignment with HugZ and the HugZ structure as a template, the resulting model will be inaccurate.

## Competing interests

The authors declare that they have no competing interests.

## Accession codes

The atomic coordinates and structure factors have been deposited in the Protein Data Bank (http://www.rcsb.org/pdb/) with the accession code 3TGV.

## Authors’ contributions

LG designed the study. XL and JG participated in the design of the study, carried out all experiments in molecular biology, protein chemistry, structure refinement, and drafted the manuscript. JG and TW performed the bioinformatic analysis. ZW and QD synthesized proteins. DZ and YH collected the X-ray data. XL and JG should be regarded as joint first authors. All authors read and approved the final manuscript.

## Supplementary Material

Additional file 1 ** Chromatographic analysis of HutZ and two unpublished proteins A and B.** Both proteins A and B exist as monomer in solution. The elution volume for A (34.7 KD) and B (17.5 KD) are 14.9 mL and 17.9 mL, respectively. The elution volume of HutZ (15 KD) was 15.7 mL. These results indicate that HutZ exists as dimer. Click here for file

Additional file 2 Greek key topology of β-barrels of HutZ and HugZ. Click here for file

Additional file 3 ** Sequence alignment of HutZ with its homologous proteins.** Vc, *Vibrio cholerae* HutZ; As, *Aliivibrio salmonicida* HuvZ; Pss, *Photobacterium* sp*.* SKA34 HugZ; Va, *Vibrio alginolyticus* protein V12G01-06051; Ah, *Aeromonas hydrophila* HutZ; Hi, *H. influenza* protein HI0854; Cj, *C. jejuni* ChuZ; Hp, *H. pylori* HugZ. Secondary structures of Vc-HutZ are schematically represented above the sequences. Residues from HutZ that coordinate the iron atom in heme are labeled with asterisks at the top; residues stabilizing the heme molecule in HutZ are labeled with squares at the top; residues from HugZ coordinating the iron atom in heme are labeled with rhombus at the bottom; residues from HugZ involved in stabilization of heme molecule are labeled with triangles at the bottom. Strictly conserved residues are marked with red background. Similar residues are shown in red color. Click here for file

Additional file 4 ** An Fo_Fc omit map calculated at 2.0 Å resolution (contoured at 1.0 sigma) for the four residues making up the β6 corner of HutZ.** The electron density map shown in gray well matches the corresponding structure, indicating the reliability of this structure. Click here for file

Additional file 5 ** Different hydrogen bonding patterns in HutZ β6 and HugZ β9.** (A) Hydrogen bonding patterns in HutZ β6. Blue sphere: nitrogen atom; red sphere: oxygen atom; green dotted line: hydrogen bond. (B) Hydrogen bonding patterns in HugZ β9. (C) Sequence correspondence based on sequence alignment. Secondary structures are labeled above or under the sequences. Hydrogen bond providing residues are in bold. From the corners up, the hydrogen bonding patterns become irregular. In β6-2 and β9-2, the hydrogen bond providing residues mismatch by one amino acid residue. Click here for file
