# Supplementary figures and images for: Crystal structure of HutZ, a heme storage protein from Vibrio cholerae: A structural mismatch observed in the region of high sequence conservation
Source: BMC Struct Biol. 2012 Sep 26;12:23. doi: 10.1186/1472-6807-12-23 (PMC3472187; doi:10.1186/1472-6807-12-23)

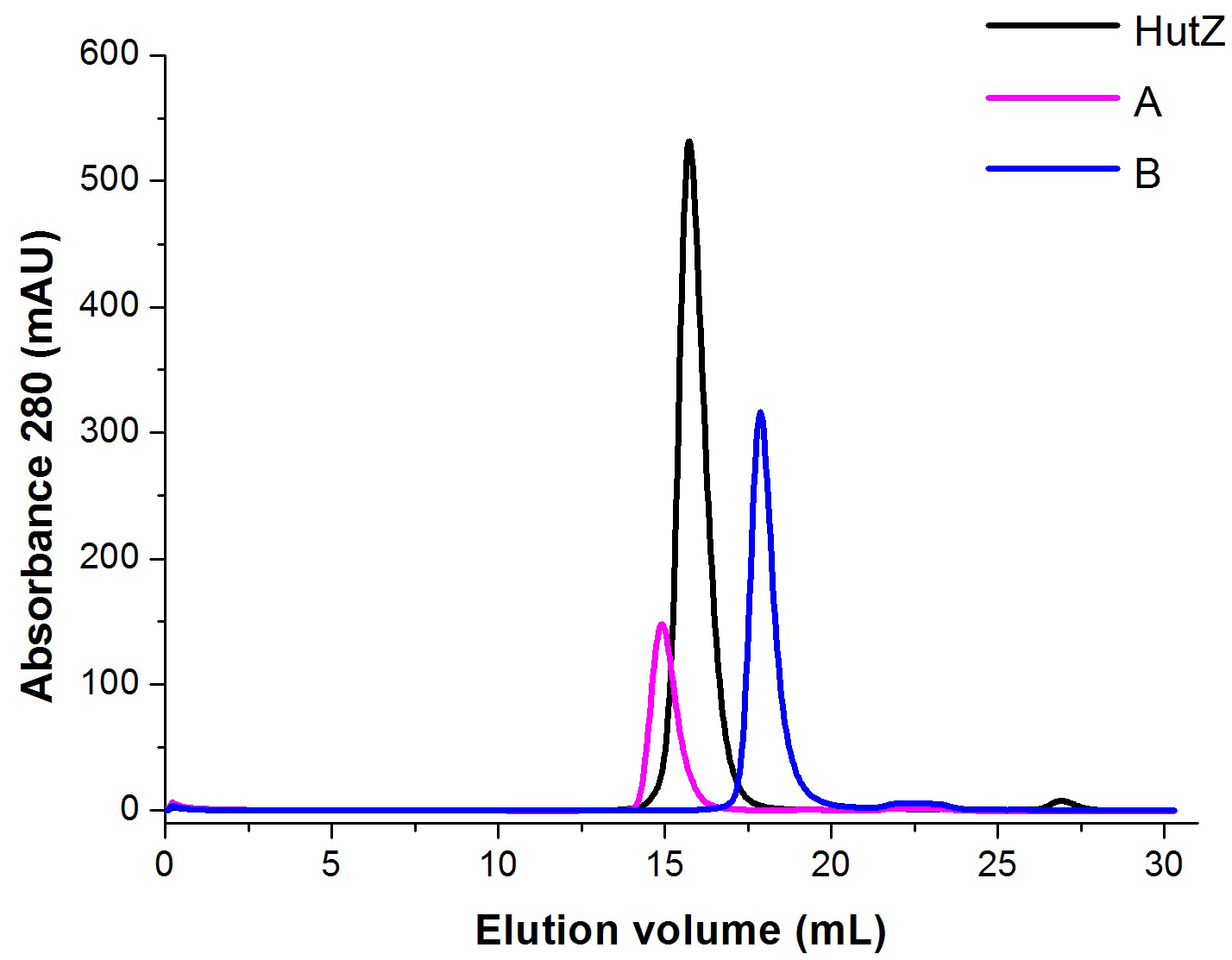

Supplement: Additional file 1 — Chromatographic analysis of HutZ and two unpublished proteins A and B. Both proteins A and B exist as monomer in solution. The elution volume for A (34.7 KD) and B (17.5 KD) are 14.9 mL and 17.9 mL, respectively. The elution volume of HutZ (15 KD) was 15.7 mL. These results indicate that HutZ exists as dimer. [file 1472-6807-12-23-S1.tiff]

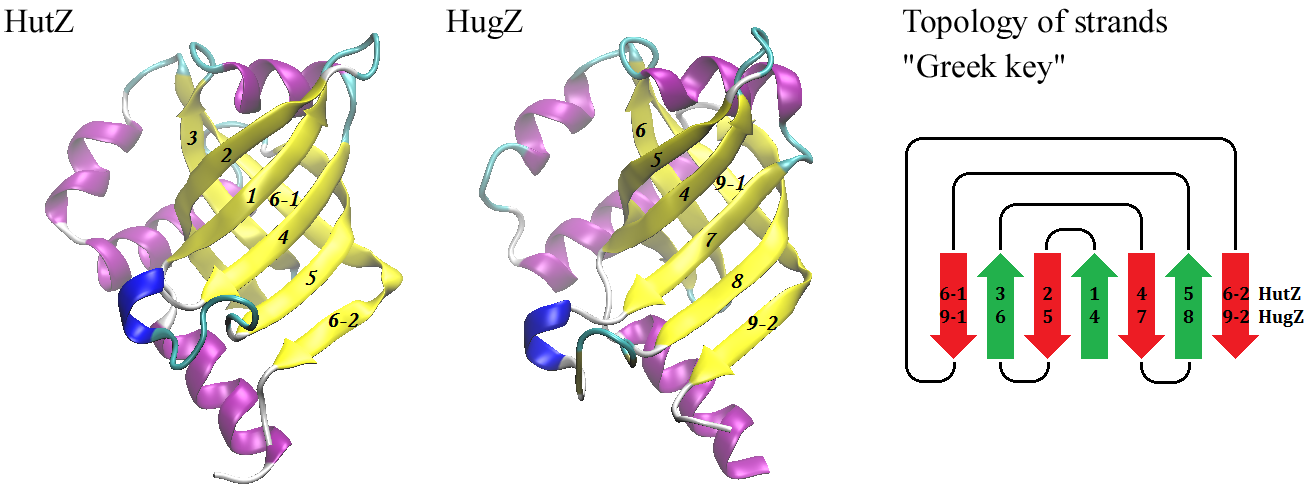

Supplement: Additional file 2 — Greek key topology of β-barrels of HutZ and HugZ. [file 1472-6807-12-23-S2.tiff]

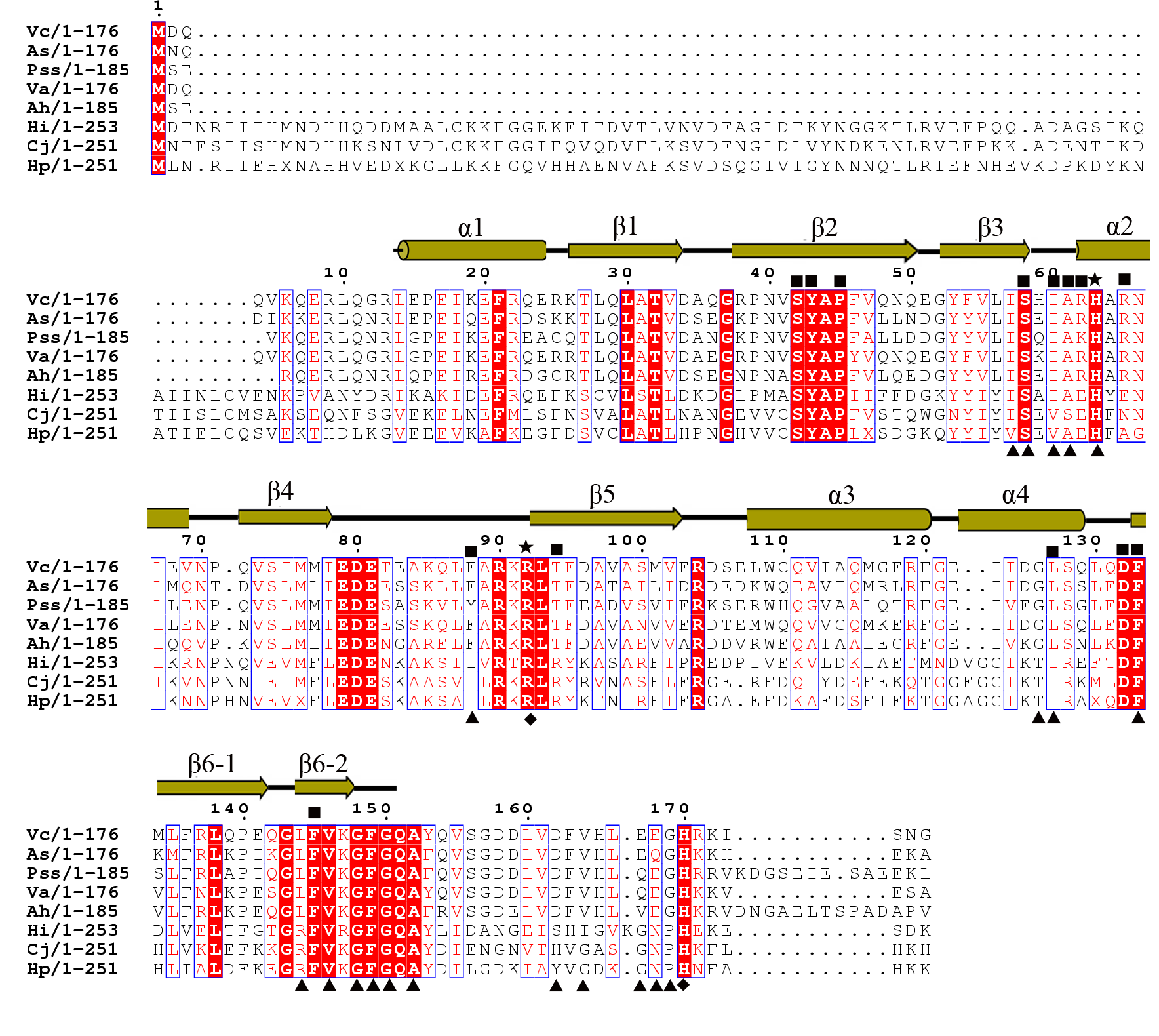

Supplement: Additional file 3 — Sequence alignment of HutZ with its homologous proteins. Vc, Vibrio cholerae HutZ; As, Aliivibrio salmonicida HuvZ; Pss, Photobacterium sp. SKA34 HugZ; Va, Vibrio alginolyticus protein V12G01-06051; Ah, Aeromonas hydrophila HutZ; Hi, H. influenza protein HI0854; Cj, C. jejuni ChuZ; Hp, H. pylori HugZ. Secondary structures of Vc-HutZ are schematically represented above the sequences. Residues from HutZ that coordinate the iron atom in heme are labeled with asterisks at the top; residues stabilizing the heme molecule in HutZ are labeled with squares at the top; residues from HugZ coordinating the iron atom in heme are labeled with rhombus at the bottom; residues from HugZ involved in stabilization of heme molecule are labeled with triangles at the bottom. Strictly conserved residues are marked with red background. Similar residues are shown in red color. [file 1472-6807-12-23-S3.tiff]

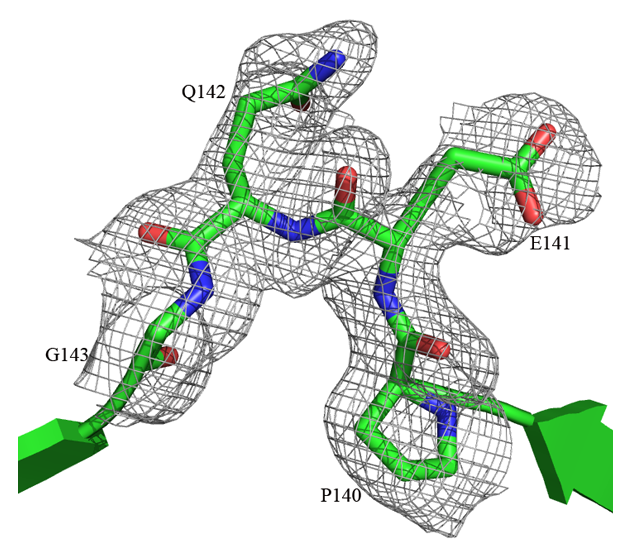

Supplement: Additional file 4 — An Fo_Fc omit map calculated at 2.0 Å resolution (contoured at 1.0 sigma) for the four residues making up the β6 corner of HutZ. The electron density map shown in gray well matches the corresponding structure, indicating the reliability of this structure. [file 1472-6807-12-23-S4.tiff]

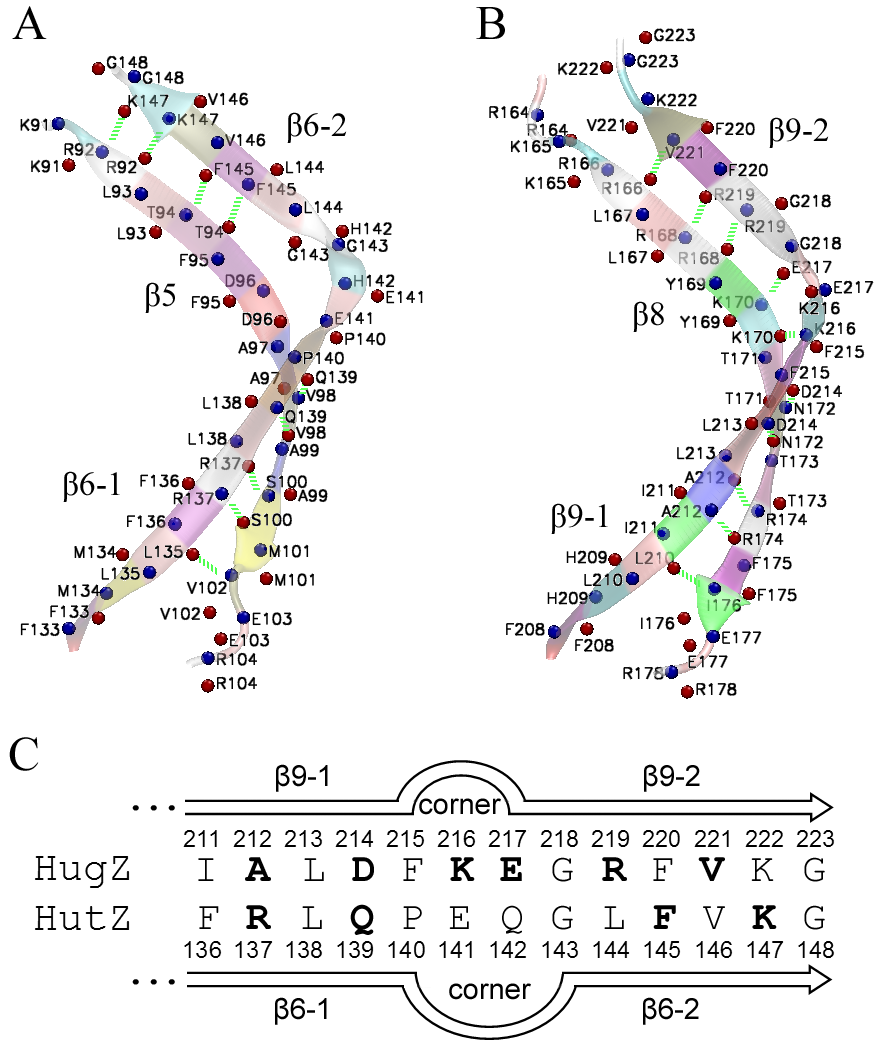

Supplement: Additional file 5 — Different hydrogen bonding patterns in HutZ β6 and HugZ β9. (A) Hydrogen bonding patterns in HutZ β6. Blue sphere: nitrogen atom; red sphere: oxygen atom; green dotted line: hydrogen bond. (B) Hydrogen bonding patterns in HugZ β9. (C) Sequence correspondence based on sequence alignment. Secondary structures are labeled above or under the sequences. Hydrogen bond providing residues are in bold. From the corners up, the hydrogen bonding patterns become irregular. In β6-2 and β9-2, the hydrogen bond providing residues mismatch by one amino acid residue. [file 1472-6807-12-23-S5.tiff]
